# Work–Family Conflict and Mental Health among Chinese Female Healthcare Workers during the COVID-19 Pandemic: The Moderating Effects of Resilience

**DOI:** 10.3390/healthcare11121696

**Published:** 2023-06-09

**Authors:** Zixiao Liu, Liu Hong

**Affiliations:** 1 School of Humanities, Tongji University, Shanghai 200092, China; 2 School of Social Development and Public Policy, Fudan University, Shanghai 200433, China

**Keywords:** COVID-19, female healthcare workers, mental health, work–family conflict, resilience

## Abstract

(1) Background: The mental health of female healthcare workers is at greater risk during the COVID-19 pandemic due to increased psychological stress and heightened work–family conflict. This study investigated whether resilience, as a protective factor for mental health, can safeguard the well-being of female healthcare workers. This study assessed the mental health of female healthcare workers (*n* = 431) from a small inland city in Central China, explored the impact of work–family conflict on their mental health, and examined the moderating role of resilience. (2) Methods: The main variables were measured using standard tools administered via an online survey. A one-sample *t*-test, ANOVA, Pearson correlation analysis, and multiple regression were performed with SPSS. A simple slope test was conducted based on the multiple regression results. (3) Results: The analysis revealed that the mental health level of the surveyed female healthcare workers was significantly lower than the national norm (t = 16.36, *p* < 0.001). Work–family conflict had a significant negative impact on mental health (β = 0.39, *p* < 0.001), while the interaction effect of resilience and work–family conflict was significant (β = −0.13, *p* < 0.05), suggesting a moderating effect. (4) Conclusions: Female healthcare workers exhibited poor mental health during the COVID-19 pandemic, but resilience remained a protective factor, mitigating the negative impact of work–family conflict on female healthcare workers’ mental health.

## 1. Introduction

Since the outbreak of the COVID-19 pandemic, healthcare professionals have been on the front lines tackling the global public health emergency. China’s strict infection prevention and control policies spanned nearly three years, from 2020 to early 2023. This prolonged workload has subjected medical practitioners to sustained professional and psychological strain [1,2]. Numerous studies have revealed that healthcare workers are susceptible to mental health issues such as depression, anxiety, and insomnia [3,4,5,6,7]. Owing to China’s vast population, the ratio of healthcare workers to the total population is only 0.99%, placing it at the midpoint globally and considerably lower than developed nations in Europe and the United States [8,9]. Consequently, the work intensity of Chinese healthcare workers, in light of the COVID-19 pandemic, has escalated significantly. A previous study in China reported that half of frontline nurses experienced moderate to high levels of burnout [10], further exacerbating the risk of psychological disorders among healthcare professionals [11]. Studies from China and other countries have reported a higher prevalence of mental health symptoms among female healthcare workers compared to their male counterparts [12,13,14,15]. Research suggests that the elevated incidence of mental health problems among female healthcare workers during the COVID-19 pandemic may be attributable to the multiple psychological stressors they face.

Female healthcare workers have been exposed to a demanding workload, especially nurses, who carry a higher caregiving burden and increased exposure risk [16]. Studies have found that female nurses in similar positions have a higher rate of depression than male nurses [17]. Even nursing students are facing more work and infection risks due to the impact of the COVID-19 pandemic [18,19]. When work intensity increases, work–family conflict becomes more intense. Research has shown that the longer weekly working hours of nurses predict more intense work–family conflict [20]. Large-sample quantitative research indicated that the intensity of nurses′ work, their daily working hours, and the pressure to work overtime emerged as important predictors of work–family conflict [21]. A qualitative study undertaken during the COVID-19 pandemic in China revealed a notable surge in the workload of healthcare workers. Following their shifts, they frequently retreated directly to their residences, experienced significant exhaustion, and exhibited a lack of motivation for further movement [22]. Many healthcare workers operated without respite due to pandemic prevention and control measures, with over a quarter persistently working more than eight hours a day. Prolonged and uninterrupted work hours significantly heightened the risk of burnout [23]. As a result, many female healthcare workers struggled to balance their professional and family roles. They had to stay at their posts during the critical moments of the pandemic while also assuming family caregiving roles.

Previous studies have shown that married female nurses experience intense work–family conflict, have shorter sleep durations than single nurses, and are more likely to develop depressive emotions [24], and working women report more mental health problems than men [25]. Meanwhile, in China, women are given more responsibility for family care due to the cultural emphasis on family and traditional gender roles [26]. Previous studies have shown that work–family conflict predicts depression in Chinese professional women [27] and negatively affects their psychological health levels [28]. Therefore, during the COVID-19 pandemic, the mental health of Chinese female healthcare workers is likely to be negatively affected by increased work–family conflict.

It is therefore important to explore protective factors contributing to the mental health of female healthcare workers in the COVID-19 pandemic, during which increased work intensity and work–family conflict may result in significant negative mental health effects. Prior research has examined the protective roles of factors such as person–environment fit, social support, mindfulness, and interpersonal relationship quality in the mental health of female healthcare workers [29,30,31,32]. Resilience is highly regarded by scholars as a crucial psychological capability that enables individuals to manage their mental health and recover from adversity when facing stress, challenges, and major events such as natural disasters, political violence, and epidemics [33,34,35,36]. Empirical research has demonstrated the role of resilience as a protective factor for mental health under various circumstances. This includes the moderation of the impact of exposure to war trauma on post-traumatic stress disorder among refugees [37], attenuation of the effects of stressful life events on postpartum depression in women [38], and mitigation of the adverse consequences of perceived stress on binge eating symptoms among young adult women [39]. During the COVID-19 pandemic, research has shown that resilience can still moderate negative effects on mental health for both patients and healthcare workers [40,41].

Can resilience play a similar protective role in moderating the impact of work–family conflict on the mental health of female healthcare workers? This is especially relevant during public health emergencies such as COVID-19, when female healthcare workers face greater psychological stress and work intensity than usual, leading to more intense work–family conflict. This study aimed to investigate this mechanism based on a cross-sectional survey of the mental health of Chinese female healthcare workers during the COVID-19 pandemic. It provides valuable information for alleviating the psychological pressures on female healthcare workers and improving their mental health levels during such challenging times. The study is reported in four sections: the Materials and Methods, Results, Discussion, and Conclusions.

## 2. Materials and Methods

### 2.1. Data Collection

The data reported here were drawn from an online survey of female healthcare workers conducted in a county within a small inland city in Central China between 20 September and 8 October 2022. The study received ethical approval from the Ethics Committee of the School of Humanities at Tongji University (approval date: 5 September 2022). A questionnaire link was distributed to 930 female healthcare workers in all three county hospitals. After reading an informed consent statement, female healthcare workers voluntarily participated in the survey by filling out the questionnaire. A total of 433 female healthcare workers completed the questionnaire, with a response rate of 46.6%. All responses were screened, and those that were submitted within the specified timeframe and without apparent perfunctory answers were considered valid and included for analysis. The final dataset consisted of 431 valid responses, with a 99.5% efficiency rate. 

### 2.2. Measures

#### 2.2.1. Mental Health

The respondents’ mental health was assessed using the SCL-90 (Symptom Checklist 90) [42]. This scale consists of 90 questions, with respondents rating each item on a 5-point Likert scale ranging from 1 = “none” to 5 = “severe”. Higher scores indicate poorer levels of mental health. Nine subscale average scores were calculated, measuring somatization, obsession, interpersonal sensitivity, depression, anxiety, hostility, phobic anxiety, paranoid ideation, and psychoticism. This scale is widely used for measuring mental health status globally and has demonstrated good reliability and validity among the Chinese population [43]. The instrument demonstrated high internal consistency in this study (Cronbach’s α = 0.99).

#### 2.2.2. Work–Family Conflict

The Work–Family Conflict Scale developed by Carlson et al. [44] was used in this study. It consists of 18 items divided into three dimensions: time conflict, strain conflict, and behavior conflict. The scale uses a 5-point Likert scale, with 1 = “strongly disagree” and 5 = “strongly agree”. Higher scores indicate stronger work–family conflict. The scale has been translated into Chinese and validated with good reliability and validity among the Chinese population [45]. In this study, the scale’s Cronbach’s α was 0.95, confirming its good reliability.

#### 2.2.3. Resilience

The study employed the Chinese version of the 10-item Connor–Davidson Resilience Scale, translated by Wang Song-Hui et al. [46]. The scale uses a 5-point Likert scale, with 1 = “never” and 5 = “always”. Higher scores represent better resilience. The scale’s reliability and validity have been confirmed in the Chinese population [46]. In this study, the scale maintained high reliability with a Cronbach’s α of 0.93.

### 2.3. Statistical Analysis

The data were analyzed using the SPSS v.19.0 statistical software package, with the significance level set at α < 0.05. First, descriptive statistics for the demographic variables and study variables were calculated. Next, ANOVA and post hoc tests (LSD) were conducted on the study variables, categorizing the demographic variables. Single-sample *t*-tests were then performed, comparing the mean total score and mean score of each SCL-90 dimension with the national norm [47]. Pearson’s bivariate correlation analysis was used to examine the relationships between the study variables. Finally, after centering the independent and moderating variables, moderation analysis was performed using multivariate regressions with the SPSS PROCESS 3.4.1 macro [48]. Model 1 examined the moderating effect of resilience (moderator) on the relationship between work–family conflict variables (independent variables) and mental health variables (dependent variables), controlling for age, education level, job position, marital status, and whether or not the participants had children. The simple slopes of the variables involved in the moderation were plotted based on the mean ± 1 standard deviation of the moderation variables and the independent variables using the pick-a-point approach [49].

## 3. Results

### 3.1. Description of the Sample Characteristics and Differences in Study Variables

The sample included 431 Chinese female healthcare workers. The mean age of the participants was 34.33 years (SD = 8.23 years), ranging from 20 to 56 years. The age distribution of the participants was as follows: 20–29 years (*n* = 132, 30.6%), 30–39 years (*n* = 180, 41.8%), and 40 years and above (*n* = 119, 27.6%). While there was no significant difference in mental health levels between age groups, significant differences in work–family conflict (F = 4.58, *p* < 0.001) and resilience (F = 11.96, *p* < 0.001) were found between them. The post hoc test results showed that female healthcare workers aged 20–29 years had significantly higher work–family conflict and significantly lower resilience than those aged 40 years and above (Table 1).

In terms of education level, most participants held a junior college degree (*n* = 222, 51.5%) or a bachelor’s degree (*n* = 196, 45.5%), with a small portion having a high school diploma (*n* = 13, 3%). No significant differences were found in mental health, work–family conflict, or resilience across education levels. Regarding job positions, most participants were nurses (*n* = 241, 55.9%), followed by doctors (*n* = 83, 19.3%), medical technicians (*n* = 47, 10.9%), administrative staff (*n* = 27, 6.3%), other positions (*n* = 21, 4.9%), and logistics staff (*n* = 12, 2.8%). The ANOVA and post hoc tests found no significant differences in psychological resilience scores across positions, but significant differences were found in the mental health and work–family conflict scores.

Regarding marital status, most female healthcare workers were married (*n* = 318, 73.8%), with a smaller portion being unmarried (*n* = 113, 26.2%). No significant differences were found between marital statuses in terms of mental health, work–family conflict, or resilience. Most participants had children (*n* = 308, 71.5%), while a smaller portion did not (*n* = 123, 28.5%). ANOVA showed differences between those with and without children, with female healthcare workers with children having significantly higher resilience than those without children. No significant differences were found between the two groups in terms of mental health and work–family conflict (Table 1).

### 3.2. Mental Health Status of Female Healthcare Workers during the COVID-19 Pandemic Compared with the National Norm

The SCL-90 scores of the sample in the current study were compared with the national norm for Chinese adults. This norm was established based on a survey conducted in 1986, which included 1388 adults aged 18–60 years from 13 regions in China. The sample comprised a balanced gender distribution of 724 males and 664 females and effectively represented all occupations and education levels [47]. It was found that during the COVID-19 pandemic, female healthcare workers scored significantly higher in the total score (t = 16.31, *p* < 0.001) and all nine sub-dimensions, including anxiety (t = 14.75, *p* < 0.001), depression (t = 16.23, *p* < 0.001), somatization (t = 17.67, *p* < 0.001), obsession (t = 20.07, *p* < 0.001), interpersonal sensitivity (t = 9.86, *p* < 0.001), hostility (t = 13.36, *p* < 0.001), phobic anxiety (t = 14.15, *p* < 0.001), paranoid ideation (t = 10.36, *p* < 0.001), and psychoticism (t = 14.75, *p* < 0.001), compared to the national norm (Table 2).

### 3.3. Relationship between Mental Health, Work–family Conflict, and Resilience

During the COVID-19 pandemic, female healthcare workers′ SCL-90 scores were significantly positively correlated with work–family conflict (r = 0.46, *p* < 0.01) and significantly negatively correlated with resilience (r = −0.45, *p* < 0.01). Additionally, their work–family conflict was significantly negatively correlated with psychological resilience (r = −0.32, *p* < 0.01) (Table 3).

### 3.4. Moderating Effects of Resilience 

In the moderated model, work–family conflict had a significant positive effect on the SCL-90 scores (β = 0.39, *p* < 0.001), meaning that greater work–family conflict led to higher SCL-90 scores and poorer mental health levels. The interaction between work–family conflict and resilience had a significant negative effect on the SCL-90 scores (β = −0.13, *p* < 0.05), indicating that the effect of work–family conflict on female healthcare workers′ mental health was moderated by resilience (Table 4).

**Figure 1:**
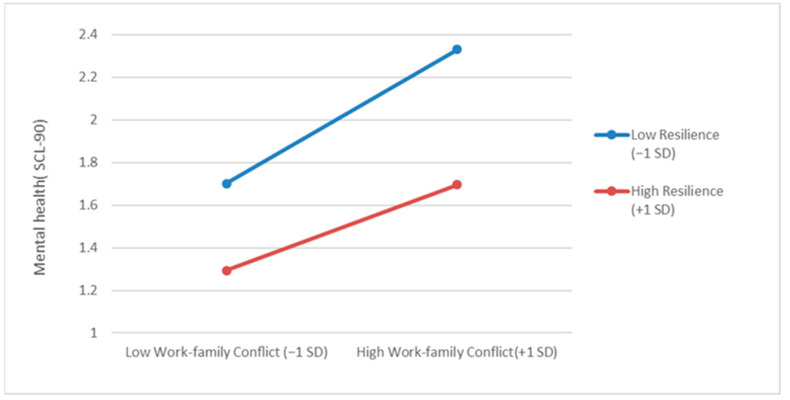
Simple slope analysis for low resilience (1 SD below the mean) and high resilience (1 SD above the mean).

The simple slope test showed that the influence of work–family conflict on mental health was weakened for female healthcare workers with high resilience (1 SD above the mean) compared to those with low resilience (1 SD below the mean), as shown in Figure 1.

Table 5 showed 95% confidence intervals for the slopes of high resilience (1 SD above the mean) and low resilience (1 SD below the mean).

## 4. Discussion

This study aimed to examine the impact of work–family conflict on the mental health status of female healthcare workers during the COVID-19 pandemic, when work intensity significantly increased, and whether this impact was moderated by resilience. The results showed that during September and October 2022, the SCL-90 scores of the female healthcare workers involved in this study were significantly higher (*p* < 0.001) than the Chinese national norm for both the total score and the nine sub-dimensions, indicating that their mental health levels were significantly lower than the Chinese national norm. For context, a 2020 study on frontline healthcare workers in Wuhan found that their SCL-90 scores were higher than the Chinese national norm in six sub-dimensions [50], while a study of healthcare workers in the southern city of Zhuhai in July 2020, when China’s pandemic prevention and control measures reached a stage of stability, found that their SCL-90 scores in all nine sub-dimensions were significantly lower than the Chinese national norm [51]. These findings suggest that the mental health of female healthcare workers may have worsened when new waves or variants of the virus emerged, such as the Omicron variant, due to prolonged and intense pandemic prevention and control measures, as chronic emotional stress can lead to poor mental health [52].

The subgroup comparison found significant differences between age groups and between job positions. Female healthcare workers aged 40 and above had significantly lower work–family conflict and were more resilient than those in the 20–29 and 30–39 age groups. This may be due to the fact that in Chinese families, women’s status within the family increases with age. The Chinese saying “a daughter-in-law becomes a mother-in-law” means that women’s status within the family significantly improves as they age, especially in areas with strong traditional cultural influences [53]. Therefore, in the area explored in this study, where traditional Chinese culture is strongly influential, female healthcare workers over 40 should have higher family status and fewer family tasks, thus reducing their work–family conflict. Higher family status also means that they are more respected, cared for, and supported within the family, which are considered protective factors for resilience [54,55]. The cultural tradition of grandparental care in China [56] may help to reduce work–family conflict for female healthcare workers over 40, as their retired parents are more likely to provide valuable support in managing childcare and other family responsibilities, potentially improving healthcare workers′ well-being during challenging times. In addition, more work experience may also render older female healthcare workers more comfortable with stressful situations [53], contributing to their higher levels of resilience. In terms of position, nurses had significantly higher SCL-90 scores and work–family conflict levels than doctors, which is consistent with previous findings [57,58]. This suggests that female nursing workers, who bear more caregiving burdens, need more support and help with their mental health and work–family conflict. 

Regarding the relationship between mental health, work–family conflict, and resilience, the SCL-90 scores were significantly positively correlated with work–family conflict at a moderate level (r = 0.46, *p* < 0.01) and negatively correlated with resilience at a moderate level (r = −0.45, *p* < 0.01). This indicates that increased work–family conflict among female healthcare workers is accompanied by decreased mental health levels, while increased resilience levels are accompanied by increased mental health levels. Work–family conflict was significantly negatively correlated with mental health, which is consistent with the findings of several previous studies: a 2018 study of Chinese female employees showed that women’s perceptions of work–family conflict were significantly negatively correlated with their mental health [28]. An Australian longitudinal study also revealed that parents who endured long-term work–family conflict reported the worst mental health status, while both mothers’ and fathers′ mental health improved significantly when work–family conflict was reduced [59]. Numerous studies, both cross-sectional and longitudinal, have demonstrated a significant positive relationship between resilience and mental health. This holds true across different demographic groups, including nursing professionals [60], college students [61], psychiatric patients [62], and the elderly [63]. In all these studies, populations with higher resilience were found to have superior mental health outcomes and fewer negative emotional states, such as anxiety, depression, and loneliness.

The moderated model further showed a significant interaction between resilience and work–family conflict (β = −0.13, *p* < 0.05), and a further simple slope test found that resilience effectively attenuated the negative effect of work–family conflict on mental health levels. This suggests that resilience can protect an individual’s mental health state and mitigate the negative effects of external stress and internal conflict on mental health, even in the continuous high-intensity stress situations caused by the COVID-19 pandemic. As previous studies have found, resilience remains an important protective factor for mental health even in the face of major unexpected events [40,41].

This also suggests that the negative effects of work–family conflict on the mental health of female healthcare workers caused by intense work stress can be reduced by strengthening one’s resilience. Existing research has demonstrated that psychological resilience can be effectively bolstered through strategies such as establishing a robust support system [64], participating in professional-led and mutual help groups [65,66], enhancing self-efficacy [67], joining stress management and resilience training programs [68], and eliciting the relaxation response as a countermeasure to stress [69]. Hospital and government health department administrators, family members, and healthcare workers themselves could adopt one or more of these approaches to improve resilience, thus protecting the mental health of female healthcare workers even in situations of elevated psychological stress and work–family conflict during public health emergencies such as the COVID-19 pandemic.

This study has several limitations. Firstly, its cross-sectional design prevents us from drawing causal inferences; a longitudinal design could better examine the changes in female healthcare workers′ mental health and the causal relationships between mental health, work–family conflict, and resilience. Secondly, since the study location was in a small inland city influenced by traditional Chinese culture and economically underdeveloped, the findings cannot be generalized to reflect the work–family conflict of female healthcare workers in larger, more modernized Chinese cities, where Chinese women’s family status has significantly improved [70]. Thirdly, while the sample quality was good, the analysis could have benefited from a larger sample size. Fourthly, during the COVID-19 pandemic, the participation of female healthcare workers in this study was limited due to their busy schedules and reluctance to take on additional tasks. This resulted in a relatively low response rate (46.6%), potentially leading to some degree of sampling bias. Future studies should conduct longitudinal research on the moderating mechanisms of resilience in regard to the effects of work–family conflict on mental health among female healthcare workers and include larger and representative samples to reflect different regions of China.

Despite these limitations, this study provides valuable data on factors influencing the mental health of female healthcare workers during the COVID-19 pandemic and protective moderators such as resilience. It is one of the first studies to examine the relationship between mental health, work–family conflict, and the moderators of conflict among female healthcare workers in this region, bearing implications for policy making by local government health departments and hospitals.

## 5. Conclusions

In summary, the results showed that the mental health level of female healthcare workers in this study was significantly lower than the national norm during the COVID-19 pandemic. However, resilience remained a protective factor for the mental health of female healthcare workers and could reduce the negative effects of intense work–family conflict on mental health. This suggests that during public health emergencies such as the COVID-19 pandemic, more attention should be paid to the work–family conflict and mental health of female healthcare workers, and appropriate measures should be taken to improve their resilience in order to protect their mental health.

## Figures and Tables

**Table 1:** A comparison of mental health (SCL-90), work–family conflict, and resilience based on demographic data.

Variables	Categories	*n* (%)	Mental Health (SCL-90)		Work–family Conflict		Resilience	
			M ± SD	F	M ± SD	F	M ± SD	F
Age(year)	20–29 (a)	132 (30.6)	2.03 ± 0.75	1.48	2.77 ± 0.73	4.58 *** a = b > c	3.30 ± 0.64	11.96 *** a = b < c
	30–39 (b)	180 (41.8)	2.05 ± 0.75		2.84 ± 0.64		3.37 ± 0.64	
	≥40 (c)	119 (27.6)	1.91 ± 0.64		2.61 ± 0.59		3.66 ± 0.63	
Educational level	High school	13 (3)	2.00 ± 0.64	0.49	2.50 ± 0.60	2.27	3.63 ± 0.56	0.81
	Junior college	222 (51.5)	1.97 ± 0.73		2.72 ± 0.63		3.44 ± 0.65	
	Bachelor	196 (45.5)	2.04 ± 0.72		2.82 ± 0.70		3.40 ± 0.67	
Position	Doctor (a)	83 (19.3)	1.87 ± 0.65	3.06 * a = e < b e < d	2.66 ± 0.72	4.38 ** e < a = c < b	3.44 ± 0.67	1.84
	Nurse (b)	241 (55.9)	2.09 ± 0.75		2.87 ± 0.63		3.35 ± 0.63	
	Medical technicians (c)	47 (10.9)	1.87 ± 0.71		2.57 ± 0.67		3.57 ± 0.73	
	Administrative staff (d)	27 (6.3)	2.17 ± 0.61		2.69 ± 0.62		3.57 ± 0.68	
	Other positions (e)	21 (4.9)	1.64 ± 0.56		2.35 ± 0.45		3.61 ± 0.63	
	Logistics staff (f)	12 (2.8)	2.10 ± 0.77		2.77 ± 0.75		3.60 ± 0.53	
Marital status	Married	318 (73.8)	1.98 ± 0.70	2.07	2.73 ± 0.64	1.26	3.45 ± 0.66	2.12
	Single	113 (26.2)	2.09 ± 0.78		2.82 ± 0.73		3.35 ± 0.63	
Whether or not the participant has children	Yes	308 (71.5)	1.99 ± 0.70	0.51	2.74 ± 0.64	0.51	3.48 ± 0.65	6.69 *
	No	123 (28.5)	2.04 ± 0.76		2.79 ± 0.71		3.30 ± 0.64	

Note: * *p* < 0.05, ** *p* < 0.01, *** *p* < 0.001.

**Table 2:** SCL-90 score in this study compared with the national norm.

Dimension	This Study (*n* = 431)	National Norm (*n* = 1388)	t
Total score	2.01 ± 0.72	1.44 ± 0.43	16.31 ***
Anxiety	1.94 ± 0.77	1.39 ± 0.43	14.75 ***
Depression	2.13 ± 0.81	1.50 ± 0.59	16.23 ***
Somatization	2.01 ± 0.75	1.37 ± 0.48	17.67 ***
Obsessive	2.37 ± 0.78	1.62 ± 0.58	20.07 ***
Interpersonal sensitivity	2.02 ± 0.78	1.65 ± 0.51	9.86 ***
Hostility	2.00 ± 0.80	1.48 ± 0.56	13.36 ***
Phobia anxiety	1.73 ± 0.73	1.23 ± 0.41	14.15 ***
Paranoid ideation	1.80 ± 0.73	1.43 ± 0.57	10.36 ***
Psychoticism	1.82 ± 0.74	1.29 ± 0.42	14.75 ***

Note: *** *p* < 0.001.

**Table 3:** Correlation between mental health, work–family conflict and resilience.

Variable	1	2	3
1. Mental health (SCL-90)	1		
2. Work–family conflict	0.46 **	1	
3. Resilience	−0.45 **	−0.32 **	1

Note: ** *p* < 0.01.

**Table 4:** Regression results for the moderating effect of resilience on the role of work–family conflict in mental health.

The Regression Equation		Overall Fitting Index		Significance of Regression Coefficient	
Outcome variable	Prognosis variate	R^2^	F	β	t
Mental health (SCL-90)	Work–family conflict	0.33	25.96 ***	0.39	8.42 ***
	Resilience			−0.40	−8.44 ***
	Work–family conflict * Resilience			−0.13	−2.36 *
	Age			0.05	1.09
	Education level			0.00	0.01
	Position			0.02	0.83
	Marital status			0.13	1.32
	Whether or not participant has children			−0.06	−0.61

Note: * *p* < 0.05, *** *p* < 0.001.

**Table 5:** Effects of work–family conflict on mental health (SCL-90) at low (1 SD below the mean) and high (1 SD above the mean) levels of resilience.

Level of Moderator Variable	*B*	SE	t	LLCI	ULCI
Low resilience	−0.65	0.06	7.78 ***	0.36	0.60
High resilience	0.65	0.06	5.35 ***	0.19	0.41

Note: *** *p* < 0.001.

## Data Availability

The data presented in this study are available upon request from the corresponding author.

## References

[1] He G., Chen Y., Wang D., Wang H. (2023). Influencing factors of work stress of medical workers in clinical laboratory during COVID-19 pandemic: Working hours, compensatory leave, job satisfaction. Front. Public Health.

[2] Liu C.Y., Yang Y.Z., Zhang X.M., Xu X.Y., Dou Q.L., Zhang W.W., Cheng A.S. (2020). The Prevalence and Influencing Factors in Anxiety in Medical Workers Fighting COVID-19 in China: A Cross-Sectional Survey. Epidemiol. Infect..

[3] Greenberg N., Docherty M., Gnanapragasam S., Wessely S. (2020). Managing mental health challenges faced by healthcare workers during COVID-19 pandemic. BMJ.

[4] Spoorthy M.S., Pratapa S.K., Mahant S. (2020). Mental health problems faced by healthcare workers due to the COVID-19 pandemic–A review. Asian J. Psychiatr..

[5] Thatrimontrichai A., Weber D.J., Apisarnthanarak A. (2021). Mental health among healthcare personnel during COVID-19 in Asia: A systematic review. J. Formos. Med. Assoc..

[6] Chatzittofis A., Karanikola M., Michailidou K., Constantinidou A. (2021). Impact of the COVID-19 Pandemic on the Mental Health of Healthcare Workers. Int. J. Environ. Res. Public Health.

[7] Carmassi C., Dell’Oste V., Bui E., Foghi C., Bertelloni C.A., Atti A.R., Buselli R., Di Paolo M., Goracci A., Malacarne P. (2022). The interplay between acute post-traumatic stress, depressive and anxiety symptoms on healthcare workers functioning during the COVID-19 emergency: A multicenter study comparing regions with increasing pandemic incidence. J. Affect. Disord..

[8] National Health and Wellness Commission China’s Health and Health Care Development Statistics Bulletin. http://www.gov.cn/xinwen/2022-07/12/content_5700670.htm.

[9] World Health Organization World Health Statistics 2020: Monitoring Health for The Sdgs, Sustainable Development Goals. World Health Organization. https://apps.who.int/iris/handle/10665/332070.

[10] Hu D., Kong Y., Li W., Han Q., Zhang X., Zhu L.X., Wan S.W., Liu Z., Shen Q., Yang J. (2020). Frontline nurses’ burnout, anxiety, depression, and fear statuses and their associated factors during the COVID-19 outbreak in Wuhan, China: A large-scale cross-sectional study. E Clin. Med..

[11] Zhou Y., Wang W., Sun Y., Qian W., Liu Z., Wang R., Qi L., Yang J., Song X., Zhou X. (2020). The prevalence and risk factors of psychological disturbances of frontline medical staff in china under the COVID-19 epidemic: Workload should be concerned. J. Affect. Disord..

[12] Lai J.B., Ma S., Wang Y., Cai Z., Hu J., Wei N., Wu J., Du H., Chen T., Li R. (2020). Factors associated with mental health outcomes among health care workers exposed to coronavirus disease 2019. JAMA Netw. Open.

[13] Pappa S., Ntella V., Giannakas T., Giannakoulis V.G., Papoutsi E., Katsaounou P. (2020). Prevalence of depression, anxiety, and insomnia among healthcare workers during the COVID-19 pandemic: A systematic review and meta-analysis. Brain Behav. Immun..

[14] Huang J.Z., Han M.F., Luo T.D., Zhou X.P. (2020). Mental health survey of medical staff in a tertiary infectious disease hospital for COVID-19. Zhonghua Lao Dong Wei Sheng Zhi Ye Bing Za Zhi.

[15] Buselli R., Corsi M., Baldanzi S., Chiumiento M., Del Lupo E., Dell’Oste V., Bertelloni C.A., Massimetti G., Dell’Osso L., Cristaudo A. (2020). Professional Quality of Life and Mental Health Outcomes among Health Care Workers Exposed to SARS-CoV-2 (COVID-19). Int. J. Environ. Res. Public Health.

[16] Zerbini G., Ebigbo A., Reicherts P., Kunz M., Messman H. (2020). Psychosocial burden of healthcare professionals in times of COVID-19—A survey conducted at the University Hospital Augsburg. Ger. Med. Sci..

[17] Kim M.Y., Yang Y.Y. (2021). Mental Health Status and Its Influencing Factors: The Case of Nurses Working in COVID-19 Hospitals in South Korea. Int. J. Environ. Res. Public Health.

[18] Swift A., Banks L., Baleswaran A., Cooke N., Little C., McGrath L., Meechan-Rogers R., Neve A., Rees H., Tomlinson A. (2020). COVID-19 and student nurses: A view from England. J. Clin. Nurs..

[19] Casafont C., Fabrellas N., Rivera P., Olivé-Ferrer M.C., Querol E., Venturas M., Prats J., Cuzco C., Frías C.E., Pérez-Ortega S. (2021). Experiences of nursing students as healthcare aid during the COVID-19 pandemic in Spain: A phemonenological research study. Nurse Educ. Today.

[20] Takeuchi T., Yamazaki Y. (2010). Relationship between work-family conflict and a sense of coherence among Japanese registered nurses. Jpn. J. Nurs. Sci..

[21] Simon M., Kümmerling A., Hasselhorn H.M. (2004). Work-home conflict in the European nursing profession. Int. J. Occup. Environ. Health.

[22] Liu Q., Luo D., Haase J.E., Guo Q., Wang X.Q., Liu S., Xia L., Liu Z., Yang J., Yang B.X. (2020). The experiences of health-care providers during the COVID-19 crisis in China: A qualitative study. Lancet Glob. Health.

[23] Zhang X., Wang J., Hao Y., Wu K., Jiao M., Liang L., Gao L., Ning N., Kang Z., Shan L. (2021). Prevalence and Factors Associated with Burnout of Frontline Healthcare Workers in Fighting Against the COVID-19 Pandemic: Evidence from China. Front. Psychol..

[24] Hwang E.H., Yu Y.B. (2021). Effect of sleep quality and depression on married female nurses’ work-family conflict. Int. J. Environ. Res. Public Health.

[25] Vargas-Jiménez E., Castro-Castañeda R., Agulló T.E., Medina C.R. (2020). Job Insecurity, Family Functionality and Mental Health: A Comparative Study between Male and Female Hospitality Workers. Behav. Sci..

[26] Zuo J., Bian Y. (2001). Gendered resources, division of housework, and perceived fairness—A case in urban China. J. Marriage Fam..

[27] Wang Y., Peng J. (2017). Work-Family Conflict and Depression in Chinese Professional Women: The Mediating Roles of Job Satisfaction and Life Satisfaction. Int. J. Ment. Health Addict..

[28] Zhou S.Y., Da S., Guo H., Zhang X. (2018). Work-Family Conflict and Mental Health Among Female Employees: A Sequential Mediation Model via Negative Affect and Perceived Stress. Front. Psychol..

[29] Chu L.C. (2014). The influence of perceived stress on work-family conflict and mental health: The moderating effect of person-environment fit. J. Nurs. Manag..

[30] Zhang H., Ye Z., Tang L., Zou P., Du C., Shao J., Wang X., Chen D., Qiao G., Mu S.Y. (2020). Anxiety symptoms and burnout among Chinese medical staff of intensive care unit: The moderating effect of social support. BMC Psychiatry.

[31] Lu F., Xu Y., Yu Y., Peng L., Wu T., Wang T., Liu B., Xie J., Xu S., Li M. (2019). Moderating effect of mindfulness on the relationships between perceived stress and mental health outcomes among Chinese intensive care nurses. Front. Psychiatry.

[32] Chu L.C. (2017). Impact of providing compassion on job performance and mental health: The moderating effect of interpersonal relationship quality. J. Nurs. Scholarsh..

[33] Tugade M.M., Fredrickson B.L. (2004). Resilient individuals use positive emotions to bounce back from negative emotional experiences. J. Pers. Soc. Psychol..

[34] Masten A.S. (2014). Global perspectives on resilience in children and youth. Child Dev..

[35] Liu J.J.W., Reed M., Girard T.A. (2017). Advancing resilience: An integrative, multi-system model of resilience. Personal. Individ. Differ..

[36] Hu T., Zhang D., Wang J. (2015). A meta-analysis of the trait resilience and mental health. Personal. Individ. Differ..

[37] Fino E., Mema D., Russo P.M. (2020). War trauma exposed refugees and posttraumatic stress disorder: The moderating role of trait resilience. J. Psychosom. Res..

[38] Julian M., Le H.N., Coussons-Read M., Hobel C.J., Schetter D.C. (2021). The moderating role of resilience resources in the association between stressful life events and symptoms of postpartum depression. J. Affect. Disord..

[39] Thurston I.B., Hardin R., Kamody R.C., Herbozo S., Kaufman C. (2018). The moderating role of resilience on the relationship between perceived stress and binge eating symptoms among young adult women. Eat. Behav..

[40] Pérez-Gómez H.R., González-Díaz E., Herrero M., de Santos-Ávila F., Vázquez-Castellanos J.L., Juárez-Rodríguez P., Moreno-Jiménez B., Meda-Lara R.M. (2022). The moderating effect of resilience on mental health deterioration among COVID-19 survivors in a Mexican sample. Healthcare.

[41] Gonçalves L., Sala R., Navarro J.B. (2022). Resilience and occupational health of health care workers: A moderator analysis of organizational resilience and sociodemographic attributes. Int. Arch. Occup. Environ. Health.

[42] Derogatis L.R., Lipman R.S., Covi L. (1973). SCL-90: An outpatient psychiatric rating scale—Preliminary report. Psychopharmacol. Bull..

[43] Lei X.S., Liu C.J., Jiang H. (2021). Mental health of college students and associated factors in Hubei of China. PLoS ONE.

[44] Carlson D.S., Kacmar K.M., Williams L.J. (2000). Construction and initial validation of a multidimensional measure of work–family conflict. J. Vocat. Behav..

[45] Wu S., Cao K. (2015). Abusive Supervision and Work-Family Conflict: The Mediating Role of Emotional Exhaustion. J. Hum. Resour. Sustain. Stud..

[46] Wang S.H., Wang T.J., Hsu S.T., Wang M.-H. (2017). Reliability and Validity of the Chinese Version of the Connor-Davidson Resilience Scale. Rehabil. Couns..

[47] Jin H., Wu W.Y., Zhang M.Y. (1986). Zhong Guo Zheng Chang Ren SCL-90 Ping Ding Jie Guo De Chu Bu Fen Xi [Preliminary analysis of the SCL-90 assessment results in the Chinese general population]. Chin. J. Nerv. Ment. Dis..

[48] Hayes A.F. (2017). Introduction to Mediation, Moderation, and Conditional Process Analysis: A Regression-Based Approach.

[49] Aiken L.S., West S.G. (1991). Multiple Regression: Testing and Interpreting Interactions.

[50] Wang J., Cheng Y.Q., Zhou Z., Jiang A., Guo J., Chen Z., Wan Q. (2020). Psychological status of Wuhan medical staff in fighting against COVID-19. Med. J. Wuhan Univ..

[51] Teng Y.Y., Sun Y.J., Wang T., Chen W., Wu Y., Chen M., Huang X. (2022). Results of symptom checklist-90 for medical personnel in Zhuhai. Occup. Health Damage.

[52] Caplan G. (1990). Loss, stress, and mental health. Community Ment. Health J..

[53] Lim S.L., Lim B.K. (2012). Po Xi Wen Ti: The “mother-in-law problem” navigating tradition and modernity in transforming familial relationships in the Chinese family. J. Fam. Psychother..

[54] Baruth K.E., Caroll J.J. (2002). A formal assessment of resilience: The Baruth Protective Factors Inventory. J. Individ. Psychol..

[55] Kumphfer K.L., Glantz M.D., Johnson J.L. (1999). Factors and processes contributing to resilience: The resilience framework. Resilience and Development: Positive Life Adaptations.

[56] Chen F., Liu G., Mair C.A. (2011). Intergenerational ties in context: Grandparents caring for grandchildren in China. Soc. Forces.

[57] Ning X., Yu F., Huang Q., Li X., Luo Y., Huang Q., Chen C. (2020). The mental health of neurological doctors and nurses in Hunan Province, China during the initial stages of the COVID-19 outbreak. BMC Psychiatry.

[58] Fang X.H., Wu L., Lu L.S., Kan X.H., Wang H., Xiong Y.J., Ma D.C., Wu G.C. (2021). Mental health problems and social supports in the COVID-19 healthcare workers: A Chinese explanatory study. BMC Psychiatry.

[59] Cooklin A.R., Dinh H., Strazdins L., Westrupp E., Leach L.S., Nicholson J.M. (2016). Change and stability in work-family conflict and mothers’ and fathers’ mental health: Longitudinal evidence from an Australian cohort. Soc. Sci. Med..

[60] Gao T., Ding X., Chai J., Zhang Z., Zhang H., Kong Y., Mei S. (2017). The influence of resilience on mental health: The role of general well-being. Int. J. Nurs. Pract..

[61] Wu Y., Sang Z., Zhang X.C., Margraf J. (2020). The Relationship Between Resilience and Mental Health in Chinese College Students: A Longitudinal Cross-Lagged Analysis. Front. Psychol..

[62] Verdolini N., Amoretti S., Montejo L., García-Rizo C., Hogg B., Mezquida G., Rabelo-da-Ponte F.D., Vallespir C., Radua J., Martinez-Aran A. (2021). Resilience and mental health during the COVID-19 pandemic. J. Affect. Disord..

[63] Gerino E., Rollè L., Sechi C., Brustia P. (2017). Loneliness, resilience, mental health, and quality of life in old age: A structural equation model. Front. Psychol..

[64] Masten A.S. (2014). Ordinary Magic: Resilience in Development.

[65] Gitterman A., Knight C. (2016). Promoting resilience through social work practice with groups: Implications for the practice and field curricula. J. Soc. Work. Educ..

[66] Carolyn K. (2017). Group Work with Homeless Mothers: Promoting Resilience Through Mutual Aid. Soc. Work.

[67] Cassidy S. (2015). Resilience Building in Students: The Role of Academic Self-Efficacy. Front. Psychol..

[68] Rosenberg A.R., Yi-Frazier J.P., Eaton L., Wharton C., Cochrane K., Pihoker C., Baker K.S., McCauley E. (2015). Promoting Resilience in Stress Management: A Pilot Study of a Novel Resilience-Promoting Intervention for Adolescents and Young Adults with Serious Illness. J. Pediatr. Psychol..

[69] Mehta D.H., Perez G.K., Traeger L., Park E.R., Goldman R.E., Haime V., Chittenden E.H., Denninger J.W., Jackson V.A. (2016). Building resiliency in a palliative care team: A pilot study. J. Pain Symptom Manag..

[70] Sun J.Y., Li J., Cho Y., Ghosh R., Sun J., McLean G. (2017). Women in Leadership in China: Past, Present, and Future. Current Perspectives on Asian Women in Leadership: A Cross-Cultural Analysis.

